# Here Comes Revenge: Peer Victimization Relates to Neural and Behavioral Responses to Social Exclusion

**DOI:** 10.1007/s10802-024-01227-4

**Published:** 2024-09-17

**Authors:** Sanne Kellij, Simone Dobbelaar, Gerine M.A. Lodder, René Veenstra, Berna Güroğlu

**Affiliations:** 1 TNO Perceptual and Cognitive Systems, Soesterberg, Netherlands; 2 Department of Sociology, University of Groningen, Groningen, Netherlands; 3 Department of Developmental Psychology, Leiden University, Leiden, Netherlands; 4 Leiden Institute for Brain and Cognition, Leiden University Medical Center, Leiden, The Netherlands; 5 Utrecht, Netherlands

**Keywords:** Bullying, Victimization, Social exclusion, Cyberball, fMRI, Insula

## Abstract

The aim of this study was to examine whether repeated victimization relates to differential processing of social exclusion experiences. It was hypothesized that experiences of repeated victimization would modulate neural processing of social exclusion in the insula, anterior cingulate cortex, and lateral prefrontal cortex. Furthermore, we hypothesized that repeated victimization relates positively to intentions to punish excluders. Exploratively, associations between neural processing and intentions to punish others were examined. The sample consisted of children with known victimization in the past two years (*n* = 82 (behavioral) / *n* = 73 (fMRI), 49.4% girls, *M*_*age*_ = 10.6). The participants played Cyberball, an online ball-tossing game, which was manipulated so that in the first block participants were equally included and in the second block they were excluded from play. Victimization was not related to neural activation during social exclusion, although there were indications that victimization may be related to increased insula activation during explicit exclusion. Behaviorally, repeated victimization was related to more intention to punish excluders. Neural activation during social exclusion did not predict intentions to punish excluders, but results tentatively suggested that increased insula activation during social exclusion may be related to increased intentions to punish. Together, these results provide a replication of earlier Cyberball studies and point toward differential processing of social exclusion by children who are victimized.

**Supplementary Information:**

The online version contains supplementary material available at 10.1007/s10802-024-01227-4.

## Introduction

It is no fun to be excluded. Belonging or being part of a group is a fundamental human need (Baumeister & Leary, 1995), and empirical research confirms that exclusion generally affects intrapersonal factors, such as self-esteem, sense of belonging, and mood (Hartgerink et al., 2015; Pharo et al., 2011). Having (repeated) negative social experiences with peers might significantly impact expectations from others in social interactions, especially in case of peer rejection and repeated interactions with malintent, such as in victimization (McDonald & Asher, Steven, 2018). Being victimized means being the receiver of repeated, intentionally aggressive or hurtful behavior, which can be physical (e.g., hitting) as well as relational (e.g., social exclusion), by one or more peers (Hawker & Boulton, 2000). Victimized children have higher levels of internalizing and externalizing symptoms (Reijntjes et al., 2010, 2011), are more sensitive to rejection (Calvete et al., 2018; Kellij et al., 2023; Mellin, 2012) and are prone to interpret social situations more negatively (Kellij et al., 2022). Thus, they are likely to have more emotional responses to social exclusion, even when they are excluded by unknown peers. Adverse peer experiences have also been related to increased neural sensitivity to social exclusion (Asscheman et al., 2019; Will et al., 2016b). Understanding the effects of victimization in late childhood may inform the development of bullying prevention earlier on in development. Therefore, we examined neural processing and subsequent behavioral responses to social exclusion in relation to repeated victimization experiences (i.e., over a period of two years) during the elementary school years (8–12 years).

### Social Information Processing and Peer Victimization

Social information processing (SIP) theory (Crick & Dodge, 1994) provides a framework for how social information is processed, and thus, how an individual experiences social situations such as being socially excluded. According to this theory, individuals attend to and register cues (encoding), interpret these cues together (interpretation), and form a response based on one’s goals and evaluation of effectiveness (behavioral response). This cycle is not necessarily gone through fully each time a social cue is presented, as people have a database consisting of memories, schemas and knowledge that are updated throughout each experience and can be contrived from in each SIP phase. A recent systematic review examined victimization experiences and the encoding and interpretation phase of social information processing and concluded that most evidence pointed towards a more negative or preventative social cognitive style, meaning that victimized persons are more prone to interpret (ambiguous) social cues in a more negative manner (Kellij et al., 2022). As victims seem prone to interpret social situations in more negative ways, it warrants the question whether victimization experiences are corroborated by differential neural processing and subsequent behavioral responses. A better understanding of an underlying (neurobiological) mechanism of the response to social exclusion, as well as the behavioral responses after being socially excluded, might provide insights to help tackle internalizing symptoms as well as possible future victimization.

### Neural Responses to Social Exclusion and Peer Victimization

Prior studies on the neural correlates of social exclusion show involvement of the anterior cingulate cortex (ACC), insula and various parts of the prefrontal cortex (PFC) (e.g., Eisenberger et al., 2003, Gunther Moor et al., 2012). An increasing number of studies have examined the neural correlates of social exclusion in populations of varying ages and characteristics. Recent meta-analyses corroborate the involvement of the ACC and the insula extending into vmPFC and vlPFC during the processing of social exclusion (Mwilambwe-Tshilobo & Spreng, 2021; Vijayakumar et al., 2017). Heightened activation of these regions may indicate affective (insula) and regulatory responses (ACC, parts of PFC) to an adverse social experience, such as social exclusion.

The impact of social exclusion events might be stronger for children who are victimzed, who may be hypersensitive to negative social cues, as (self-reported) victimization experiences relate to lower mood after social exclusion as assessed by the Cyberball paradigm (Lansu et al., 2017; Park et al., 2017; Ruggieri et al., 2013). Cyberball is a simple online ball-tossing game where participants are first being included and later excluded from playing by the other players. Even though only three studies have examined the link between victimization experiences and neural activity during social exclusion, their findings indicate that adolescents who are victimized (vs. non-victimized) might differ in how they process social exclusion (see Güroǧlu & Veenstra, 2021). In general, all three studies suggest that victimization related to greater activation in medial and lateral prefrontal brain regions during exclusion (vs. inclusion), including the insula/IFG region (Kiefer et al., 2021; McIver et al., 2018; Rudolph et al., 2016). Importantly, these studies have mostly examined the effects of victimization on neural processing of exclusion at later ages, leaving the influences of repeated victimization at younger ages unexplored. The prevalence of victimization is higher in elementary school, whereas the prevalence of bullying is higher in high school (Haltigan & Vaillancourt, 2014). Although victims of bullying in high school were often also victimized in elementary school (Bowes et al., 2013), the findings on victimization in high school may not necessarily generalize to elementary school.

Additionally, several studies have examined social exclusion in relation to adverse experiences with peers, measured with peer nominations. Will et al. (2016) reported increased dorsal ACC (dACC) activity during social exclusion (vs. inclusion) in long-term rejected children (who received few like nominations and many dislike nominations; *N* = 46, 27 high accepted and 17 chronic rejected adolescents of 12–15 years). Asscheman et al. (2019) did not find involvement of the dACC in long-term low preferred boys (*N* = 45, 27 low peer status and 28 high peer status boys of 8–12 years), but reported enhanced activity in bilateral dlPFC and right supramarginal gyrus extending into IPL during social exclusion (vs. inclusion). These studies provide the first indications that negative peer experiences may relate to differences in the neural processing of social exclusion in childhood and early adolescence. However, self-reported victimization may possibly capture a more intense experience than experiences of peer rejection as assessed by peer nominations of like and dislike, as it does not only include not being liked but also experiences of explicit hurtful behaviors by others. Therefore, in this study, we examine the effects of experiencing repeated victimization (i.e., over a period of two years) on the neural processing of social exclusion in late childhood.

### Behavioral Responses to Social Exclusion and Peer Victimization

Based on the SIP theory (Crick & Dodge, 1994), responses to being excluded are expected to influence future social interactions, as the updated database of the individual as well as the database of the interaction partner(s) will influence future social behavior. Having a database filled with victimization experiences, for example when they occur over a longer period of time, likely affects how children who are victimized interpret intentions of others (Kellij et al., 2022), but there is little research on how victimized children respond to perpetrators.

Studies examining behavioral responses to recent social exclusion (Cyberball) show that children tend to punish excluders when they get the opportunity (Gunther Moor et al., 2012; Will et al., 2015). Furthermore, the severity of punishment correlated positively with self-reports of the intention to hurt the other (Gunther Moor et al., 2012). However, no relation was found between prior peer rejection history of participants and prosocial behavior toward excluders (Will et al., 2016). Given that victimization may be a more intense experience than not being liked, it is possible that victimization does relate to more retaliatory behaviors following social exclusion.

Indeed, in vignette studies, where participants were asked to imagine being the protagonist in (negative) social scenarios involving provocations or ambiguous situations, victimized children reported more retaliation goals than defenders, outsiders, non-involved and followers (Camodeca & Goossens, 2005). Similar vignette research that used explicit social exclusion stories found that children who are prone to interpret hostile intent think of more aggressive responses to exclusion (Mazzone et al., 2021). Although vignette research is useful in capturing intentions related to hypothetical situations, it may rely less on automatic and emotional responses, and may not necessarily capture the real-life experience of getting rejected. To our knowledge, no study has examined repeated victimization experiences in relation to behavioral responses toward recent perpetrators of social exclusion. Assessments of intentions to punish others after social exclusion are needed to better understand (aggressive and retaliatory) behavioral responses that might follow social exclusion in victims of bullying.

### Current Study

In this study, we examined how a history of victimization experiences related to both neural activity during social exclusion as well as the intention to punish after a recent exclusion experience. To do so, we recruited participants from elementary schools that participate in an anti-bullying program where victimization is measured regularly. This provided us with self-reported victimization scores of the participants over the past two years. The participants then took part in our fMRI study which enabled us to examine repeated victimization experiences in relation to individual differences in neural responses to social exclusion. Based on SIP theory and prior findings (Rudolph et al., 2016; Will et al., 2016b), we hypothesized that repeated experiences of victimization would relate to stronger neural responses in brain regions involved in social exclusion (vs. inclusion), namely the insula, the ACC and the lPFC. Further, we hypothesized that repeated victimization experiences would positively correlate with intentions to punish excluders. In addition, in secondary analyses, we examined the effect of more recent victimization on neural and behavioral responses to social exclusion. Finally, we explored whether neural and behavioral responses to social exclusion were associated, by examining whether neural sensitivity to social exclusion would predict the intention to punish (e.g., Gunther Moor et al., 2012).

## Method

### Participants

A single cohort of 83 children who attended grades 6 to 8 of elementary schools, participated in the study (sex: 49.4% girls, *M*_*age*_ = 10.6, range 8–12 years); see https://osf.io/hmq8z for pre-registration of the larger project. Participants attended elementary schools participating in the KiVa anti-bullying program where they had at least two self-reports of victimization in the past two years (41.0% had two, 47.0% had three, 9.6% had four, and 2.4% had 5 measures of self-reported victimization before the scanning day).

During the winter of 2020–2021, all 152 schools participating in the KiVa anti-bullying program in a range of 100 km from the scanning facilities were contacted and asked to send a letter and a short video about our research project to the parents of all children in grades 6 to 8 for recruitment purposes (5 schools (3.28%) could not be reached, 104 schools (68.42%) declined participation, and 43 schools (28.29%) sent the letters). Parents could then sign up their children for participating in the study and provide us with consent to access their child’s data on bullying and victimization from prior years (168 responses: 156 permissions granted (92.8%), 10 permissions denied (6.0%), 2 no answer given (1.2%)). Among the 156 children who were signed up for participation, we invited children with at least two self-reported measures of victimization in the past two years to participate in the study at our lab, and 83 participated between January 2021 and March 2022 in our study (which is 53.2% of the signed-up children). Participants in the study came from 23 different schools.

Children were excluded if they had epilepsy, took psychotropic medications that could not be skipped for 24 h, or had MRI contra-indications (e.g., braces, metal implants). The majority of participants came from highly educated families: 25 participants (30.1%) had caretakers who both had a master’s degree or higher and 28 participants (33.7%) had caretakers who had at least a bachelor’s degree, 20 participants (24.1%) had caretakers who had finished the senior general track (or higher) in secondary school or tertiary vocational training and 10 participants (12.0%) had caretakers who had finished the vocational track of secondary school or the first 3 years of the senior general track (or higher) in secondary school.

Out of the 83 children, 10 children were excluded from the fMRI analyses due to excessive motion, and some participants had missing data on the behavioral measures due to technical errors. Therefore, 73 children (47.9% girls, *M*_age_ = 10.66, *SD* = 0.93) were included in the fMRI analyses; for analyses including behavioral measures of intentions to punish the sample size was 82 (48.8% girls, *M*_age_ = 10.65, *SD* = 0.99), for inclusion perception it was 76 (50.0% girls, *M*_age_ = 10.63, *SD* = 1.00), and for need satisfaction and mood it was 80 (47.5% girls, *M*_age_ = 10.63, *SD* = 0.99).

### Procedure

The lab visit started with an information session, where written informed consent was obtained from the parents and from participants who were 12 years old (*n* = 5). During this session, participants provided verbal assent for participation, were familiarized with the scanning environment in a mock scanner and practiced the MRI tasks (± 45 min). Afterward, the scanning session (± 60 min) took place, followed by several questionnaires and three behavioral tasks on the computer (± 70 min). The participants received a goody bag with some toys, a compensation of 50 euros and the reimbursement of travel costs. This project received ethical approval from the Medical Ethical Committee Leiden Den Haag Delft (NL71576.058.19).

### Measures

#### Victimization

Victimization was assessed twice a year in the two years prior to the lab visit, as part of the data collection within the anti-bullying intervention program at school, as well as on the day of the lab visit. The Olweus’ Bully/Victim questionnaire was used to measure victimization (Olweus, 1996). The Olweus’ Bully Victim questionnaire consists of six items. First, the questionnaire provided the participant with the definition of bullying (“Bullying is when some children repeatedly harass another child. Thus, bullying is that you are mean to someone else over and over again. It is difficult for the child who gets bullied to defend itself against this.”). Next, participants were asked to indicate how often they were bullied in the past couple of months on a 5-point scale ((1) not at all, (2) once or twice, (3) two or three times per month, (4) about once a week, or (5) several times per week). After this, participants were asked five questions about how often they were bullied in terms of specific types of victimization (i.e., verbal, physical, relational, material and online). See Supplement 1 for the questions. Victimization scores were calculated for each wave by averaging all six victimization questions. The reliability of the victimization scale for each wave and the lab visit was high (Cronbach’s alpha ranging between 0.82 and 0.91).

We used two separate victimization scores in our analyses: repeated victimization (used in the primary analyses) and recent victimization (used in secondary analyses). The repeated victimization score was calculated by averaging victimization scores of all available waves of that individual (i.e., in the two years prior to the lab visit and during the lab visit; see https://osf.io/x32g9 for the validation of this chosen method). The recent victimization score was calculated by averaging the six victimization questions administered during the lab visit. Given the uniqueness of the sample with longitudinal victimization data, we used the repeated victimization score in primary analyses. However, because recent effect may be more pronounced and the recent victimization variable showed more variation, we included recent victimization scores in secondary analyses.

#### fMRI Task

To assess neural responses to social exclusion we used the Cyberball task (Will et al., 2016; Williams et al., 2000), an online ball-throwing game with three players (programmed in Authorware 7). Every time a participant received the ball, they could throw the ball to one of the other two unfamiliar players using their right or left index finger. Each of the two blocks of the game consisted of 30 ball throws (trials). In the first ‘inclusion’ block of the game, the participants played with two (pre-programmed) includers (one boy and one girl with common names), where each player received an equal number of balls, leading to ten ball-receiving, ten non-receiving, and ten ball-throwing trials. In the second ‘exclusion’ block, the participant played with two new players who were (pre-programmed) excluders (one boy and one girl with two new names). In this second block, the participant received the first ball that was thrown, but did not receive a ball again during the remaining throws; the two excluders only threw the ball to one another and not to the participant. In the excluding block there was only one ball-receiving trial, one ball-throwing and 28 non-receiving (i.e., exclusion) trials. There were two versions of the task where the names used for the two players in the inclusion and exclusion blocks were reversed; these two versions were counterbalanced across the participants. Whether the Cyberball paradigm led to feelings of exclusion was checked with three sets of questions (see Supplement 1).

### Intention to Punish

Intention to punish the excluders was measured after the exclusion block of the game based on four questions assessed on a 5-point Likert scale (1) Entirely disagree to (5) Entirely agree (Will et al., 2016). An example question was “I would like to hurt [names of excluders]”. Responses on the four items were averaged, leading to a score of willingness to punish others that ranged between 1 and 5, with higher scores indicative of increased intentions to punish excluders (Cronbach’s α = 0.74).

### Feelings of Exclusion

Whether the Cyberball paradigm led to feelings of exclusion was checked with three sets of questions. First, inclusion and exclusion perception (Will et al., 2016) was assessed with two questions outside the scanner before the debriefing. The participants were asked whether they felt included by the other players (‘In the first/second ballgame I was being included by others’) on a 5-point Likert scale (1) Entirely disagree, to (5) Entirely agree. Higher answers indicated they felt more included in that particular Cyberball block.

Second, need satisfaction (Gunther Moor et al., 2012; Will et al., 2016b) was assessed with two questions in the scanner after the inclusion block and exclusion block and outside the scanner after debriefing that the other players were not real and that their behavior was pre-programmed. Participants were asked to answer these questions based on how they were feeling at that moment on a 5-point Likert scale (1) Entirely disagree, to (5) Entirely agree. An example question was “I feel confident” (see Supplement 1 for all questions). Average scores were calculated for the two questions of each moment of acquisition (Inclusion *r* =.25, *p* =.025; Exclusion *r* =.20, *p* =.074; Debrief *r* =.39, *p* <.001). Possible scores ranged from 1 to 5, with higher scores indicating need satisfaction.

Finally, mood (Will et al., 2016) was assessed with four questions in the scanner after the inclusion block and exclusion block and outside the scanner after debriefing. Participants were asked to indicate how they felt at that moment on a 5-point Likert scale (1) Entirely disagree, to (5) Entirely agree. An example question was “I feel happy”. Average scores were calculated for the four questions for the inclusion and exclusion blocks and after debriefing (Cronbach’s α inclusion block = 0.30, exclusion block = 0.48, debrief = 0.22). Possible scores ranged from 1 to 5, with higher scores indicative of a positive mood and lower scores of a more negative mood.

### MRI Data Acquisition

MRI scans were acquired using a 3T Philips Achieva MRI scanner with a standard whole-head coil at the Leiden University Medical Center. The scanning protocol included a localizer scan, a high-resolution 3D T1 scan for anatomical reference (TR = 7.9 ms, TE = 2.5 ms, flip angle = 8°, 155 slices, voxel size = 1.04 × 1.04 × 1.10 mm, field of view [FOV] = 250.00 × 195.83 × 170.50 mm), and functional T2* weighted gradient echo planer images (EPI) (TR = 2.2 s, TE = 30 ms, 40 transverse parallel slices of 2.75 mm, FOV = 220 × 220 × 120.72 mm, 2 discarded dummy scans at the start) during two functional runs of the Cyberball game, which was self-paced and lasted approximately 3 min each. The Cyberball game was presented on a screen behind the MRI scanner that was visible through a mirror on the head coil. To minimize head movement, we placed foam pads on both sides of the participant’s head inside the head coil and ensured that participants felt comfortable.

### fMRI Preprocessing

Image preprocessing and parts of the analyses were conducted using SPM12 software (https://www.fil.ion.ucl.ac.uk/spm/). Functional images were slice-time corrected (middle slice as reference), realigned to compensate for rigid body motion, coregistered, spatially normalized to EPI T1 templates and resampled to volumes of 3 mm cubic voxels, and smoothed with a Gaussian filter of 8 mm full-width at half maximum. All results are reported in MNI305 stereotactic space.

We analyzed the fMRI data using an event-related design within the inclusion and exclusion block (following Will, van Lier et al., 2016; see also Mwilambwe-Tshilobo & Spreng, 2021). Data were modeled as a series of zero-duration events at the onset of when the ball was thrown towards a player and convolved with a canonical hemodynamic response function (HRF). The participants had to respond each time they received the ball, otherwise the game would not continue, hence there were no invalid trials. Regressors were defined for three events– referring to receiving the ball (Ball), throwing the ball (Throw), and not receiving the ball (No Ball)– in each of the inclusion and exclusion blocks in the general linear model (GLM). The model contained a basic set of cosine functions for a high-pass filter (120 Hz). The least-squares parameter estimates of the height of the best-fitting canonical HRF for each separate condition were used in pair-wise contrasts at the subject level. The resulting images were used in higher-level group analyses. Whole-brain one-tailed *t*-tests were performed in SPM12. Whole brain analyses were conducted at the uncorrected voxel-level threshold of *p* <.001 with an FDR cluster-level correction at *p* <.05.

**Fig. 1:**
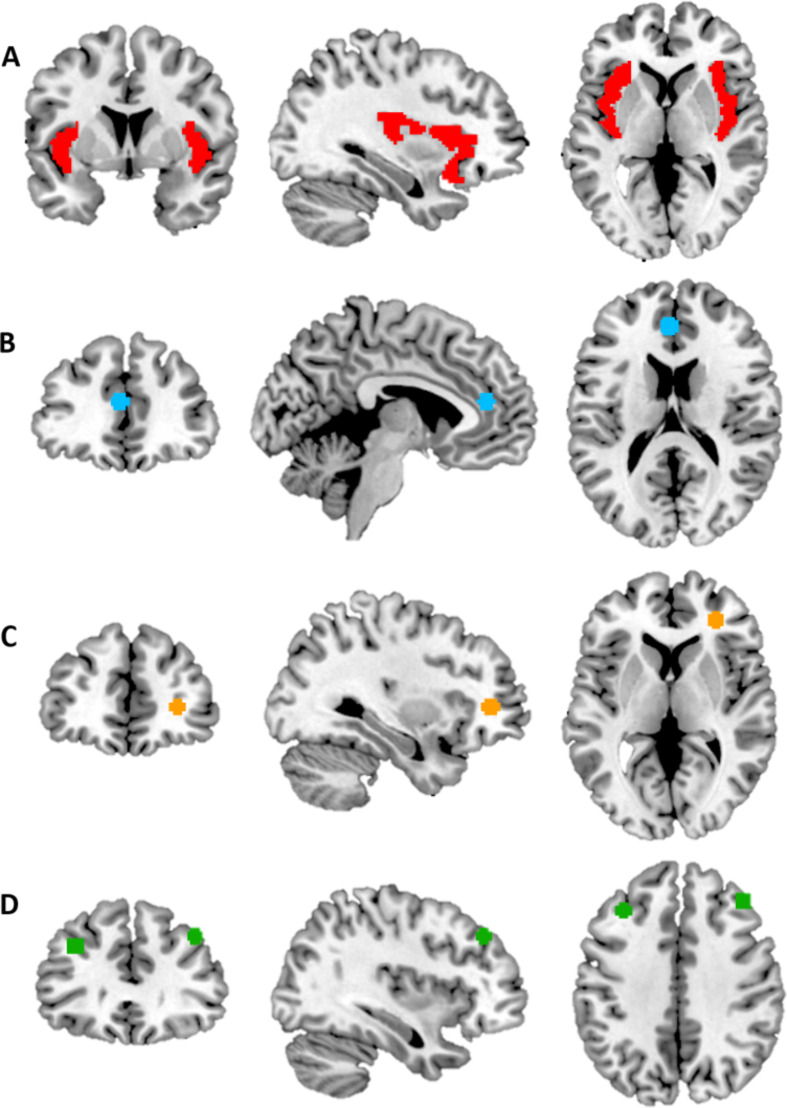
Regions of interest. **A**) bilateral insula. **B**) dorsal anterior cingulate cortex. **C**) inferior frontal gyrus. **D**) dorsolateral prefrontal cortex

We selected four independent regions of interests (ROIs; see Fig. 1), based on our preregistered hypotheses: the bilateral insula (from the Automated Anatomical Labeling Atlas (Tzourio-Mazoyer et al., 2002), the dACC (from the Exclusion No Ball > Inclusion Ball contrast in Will et al. (2016a, b), MNI peak coordinates: -3, 41, 16), the IFG (from the Exclusion > Inclusion contrast in Rudolph et al. (2016), MNI peak coordinates: 30, 41, 4), and the right and left dorsolateral prefrontal cortex (dlPFC; from the Exclusion No Ball > Inclusion No Ball contrast in Asscheman et al. (2019), MNI peak coordinates: 40, 36, 40 and − 34, 30, 34). We used the Marsbar toolbox to create 6 mm spheres around the peak coordinates (in MNI space) reported in prior studies. ROI activity values (parameter estimates) were calculated with SPM12 and exported to SPSS27 for further analysis.

### Statistical Analyses

First, to check whether the social exclusion manipulation of the Cyberball game worked, we conducted three repeated measures ANOVAs on inclusion perception, need satisfaction and mood, with assessment moment as a within-person factor (2 levels for inclusion perception: following inclusion block and exclusion block; 3 levels for need satisfaction and mood: following inclusion block, exclusion block and debriefing).

In line with confirmatory analyses outlined in our pre-registration (https://osf.io/3fhku), we analyzed three different contrasts through whole brain analyses to examine explicit exclusion, incidental exclusion and explicit exclusion vs. incidental exclusion: (1) Exclusion No Ball vs. Inclusion Ball (i.e., explicit exclusion), (2) Exclusion No Ball vs. Inclusion No Ball (i.e., explicit vs. incidental exclusion), and (3) Inclusion No Ball vs. Inclusion Ball (i.e., incidental exclusion).

In ROI analyses (insula, dACC, IFG and dlPFC), we tested whether neural activation during exclusion was related to victimization. That is, we regressed neural activation during exclusion on repeated victimization in a multivariate regression per contrast. In secondary analyses, we regressed neural activation during exclusion on recent victimization. Due to a few extreme outliers in the data, we reported the results of the analyses with the outliers adjusted to the nearest non-outlier value (Q1–1.5 × IQR & Q3 + 1.5 × IQR) to include as much data as possible. To verify that adjusting outliers did not significantly alter the results, we repeated the analyses without adjusted outliers in sensitivity analyses (see Supplement 2 and Supplementary Table S1).

For behavioral analyses, we regressed intentions to punish on repeated victimization, and recent victimization.

Finally, in exploratory analyses, we explored whether the intention to punish related to differential brain activity in the three contrasts of interest. We regressed the neural activation in the four ROIs on intention to punish in a multivariate regression per contrast.

## Results

### Descriptives

Repeated victimization ranged between 1.00 and 4.11, with a mean of 1.42 for participants included in the fMRI analyses and a mean of 1.45 for the behavioral analyses using intention to punish. Girls and boys did not differ significantly on repeated victimization from one another (*t*(81) = -1.23, *p* =.224). Repeated victimization correlated positively with recent victimization and intention to punish, but negatively with inclusion perception in the inclusion block. Intention to punish related negatively to need satisfaction after inclusion and after debriefing (see Table 1 for all means and correlations).

**Table 1:** Means and correlations

	M(SD)	*N*	RepVic	RecVic	ItP	IP1	IP2	NS1	NS2	NS3	M1	M2
Repeated victimization (RepVic)	1.46(0.53)	83										
Recent victimization (RecVic)	1.45(0.74)	85	0.77**									
Intention to punish (ItP)	2.72(1.10)	84	0.22*	0.15								
Inclusion perception (IP1)	6.10(1.47)	84	− 0.26*	− 0.09	− 0.21							
Inclusion perception (IP2)	1.88(1.75)	84	0.09	0.15	0.02	− 0.10						
Need Satisfaction (NS1)	3.86(0.84)	84	− 0.10	0.03	− 0.33*	0.20	0.01					
Need Satisfaction (NS2)	1.92(0.90)	84	− 0.06	− 0.04	− 0.01	0.01	0.15	0.07				
Need Satisfaction (NS3)	3.92(0.90)	82	− 0.20	− 0.28*	− 0.26*	0.37**	0.09	0.28*	0.21			
Mood (M1)	4.52(0.48)	84	− 0.08	− 0.10	− 0.05	0.08	0.23*	0.27*	0.18	0.18		
Mood (M2)	3.10(0.87)	84	− 0.17	− 0.14	− 0.09	0.14	0.15	0.01	0.53***	0.18	0.10	
Mood (M3)	4.56(0.43)	82	− 0.19	− 0.21	− 0.06	0.28*	0.18	0.09	0.19	0.41***	0.26*	0.19

Note^*^p <.05, ^**^p <.01, ^***^p <.001, 1 = Inclusion block, 2 = Exclusion block, 3 = After debriefing

### Manipulation Checks

#### Inclusion Perception, Need Satisfaction, and Mood

We tested whether the Cyberball manipulation worked by examining changes in inclusion perception, need satisfaction and positive mood.

On average, participants felt more included in the inclusion block than in the exclusion block (*F*(1,83) = 215.67, *p* <.001, *η*^*2*^_*p*_ = 0.72, *M*_*inclusion*_ = 6.10 ± 1.47, *M*_*exclusion*_ = 1.88 ± 1.75).

**Fig. 2:**
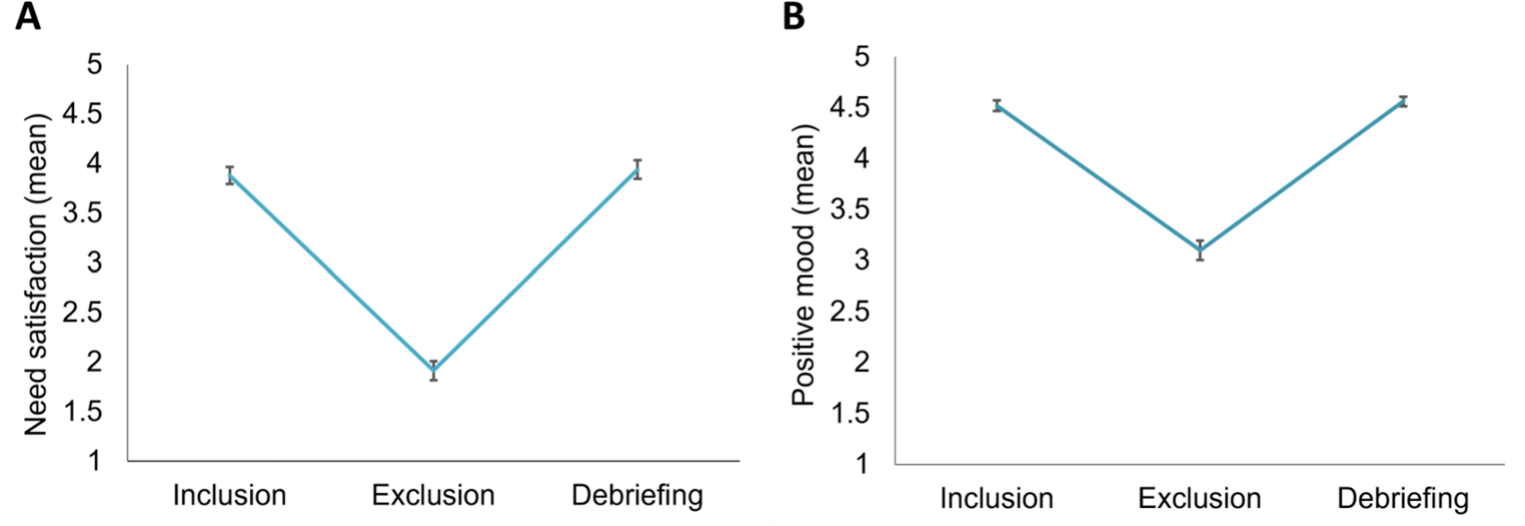
Manipulation check across the three moments of assessment: inclusion (after the inclusion block), exclusion (after the exclusion block) and debriefing (after the debriefing). **A**) scores of need satisfaction. **B**) mood. Error bars represent standard errors

A repeated measures ANOVA on need satisfaction with assessment moment as a within-person factor (3 levels: after inclusion block, exclusion block and debriefing) revealed a significant effect of assessment moment (*F*(2,160) = 179.32, *p* <.001, *η*^*2*^_*p*_ = 0.70; see Fig. 2A). Follow-up tests indicated that need satisfaction was lower in the exclusion block compared to the inclusion block and debriefing (both *p’s* < 0.001; *M*_*inclusion*_ = 3.88 ± 0.80; *M*_*exclusion*_ = 1.93 ± 0.90; *M*_*debriefing*_ = 3.94 ± 0.86).

Finally, a repeated measures ANOVA on positive mood with assessment moment as within-person factor (3 levels: after inclusion block, exclusion block and debriefing) revealed a main effect of assessment moment (*F*(2,160) = 163.72, *p* <.001, *η*^*2*^_*p*_ = 0.67; see Fig. 2B). Follow-up tests indicated that participants reported a less positive mood following the exclusion block compared to the inclusion block and debriefing (both *p’s* < 0.001; *M*_*inclusion*_ = 4.53 ± 0.47; *M*_*exclusion*_ = 3.12 ± 0.88; *M*_*debriefing*_ = 4.56 ± 0.42).

### Neural Results

#### Whole Brain Analyses

**Fig. 3:**
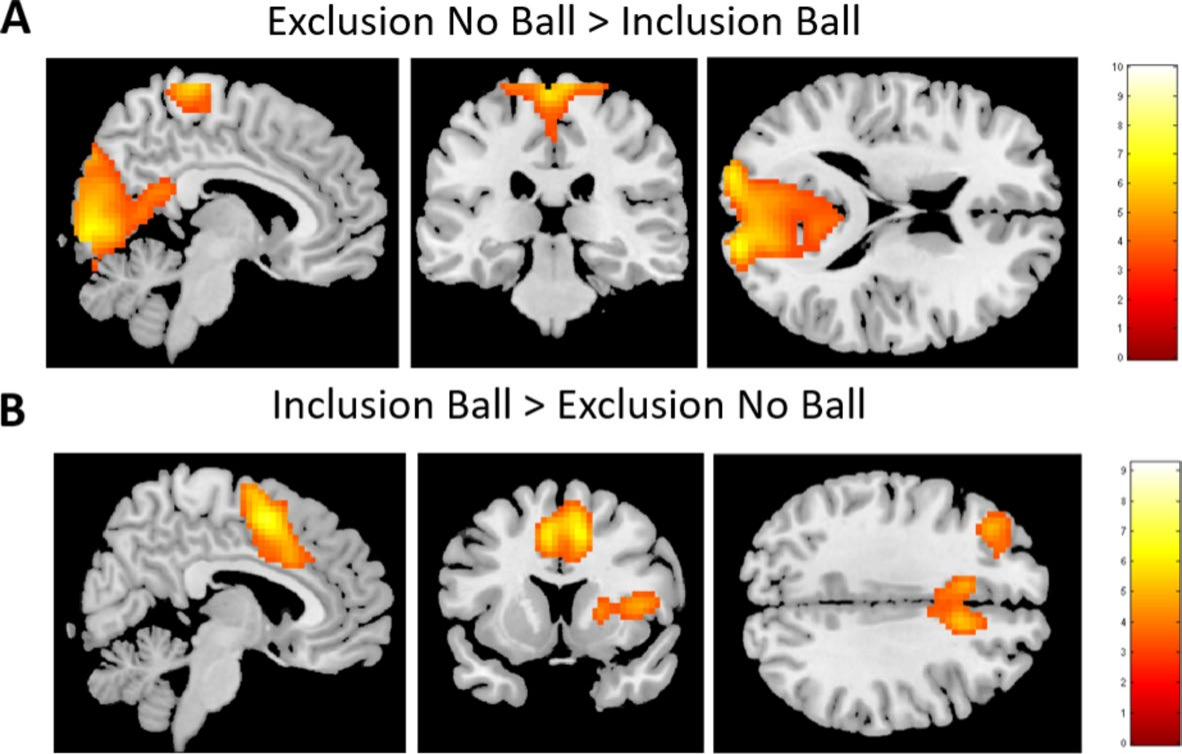
Brain activity during explicit exclusion and reversed contrast. **A**) contrast Exclusion No Ball vs. Inclusion Ball. **B**) contrast Inclusion Ball vs. Exclusion No Ball

**Exclusion No Ball vs. Inclusion Ball.** We first examined social exclusion by the whole-brain *t*-contrast for not receiving the ball (in the exclusion block) vs. receiving the ball (in the inclusion block). This contrast resulted in two clusters of activity in the occipital lobe and paracentral lobule (see Fig. 3A; Table 2).

**Table 2:** Activity in whole brain t-contrasts ‘Exclusion no ball vs. inclusion ball’ and ‘Inclusion ball vs. exclusion no ball’

Brain region	*R*/L	Cluster-level		Peak-level					
		*p* (FDR)	K	*p* (FDR)	*t*	MNI coordinates			
						x	y	z	
Explicit exclusion: Exclusion No Ball > Inclusion Ball									
***Occipital lobe***		< 0.001	2655						
Lingual gyrus	R			< 0.001	10.01	21	-91	-4	
Middle Occipital gyrus	L			< 0.001	8.92	-15	-94	-1	
Calcarine gyrus	R			< 0.001	7.91	12	-79	2	
Middle Occipital gyrus	L			< 0.001	6.52	-18	-94	17	
Cuneus	L			0.006	5.51	-3	-82	23	
***Paracentral lobule***		0.003	447						
Paracentral lobule	L			0.001	6.18	-3	-28	71	
Paracentral lobule	R			0.002	6.00	6	-28	74	
Paracentral lobule	L			0.002	5.77	-9	-37	74	
Postcentral gyrus	R			0.011	5.27	21	-31	74	
Middle cingulate cortex	L			0.099	4.38	0	-34	50	
Inclusion: Inclusion Ball > Exclusion No Ball									
***SMA***		< 0.001	1553						
Precentral gyrus	L			< 0.001	9.24	-18	-7	68	
Sup. Frontal gyrus	R			< 0.001	7.91	24	-4	68	
SMA	L			< 0.001	6.97	-6	-1	59	
SMA	R			< 0.001	6.81	9	5	53	
SMA	L			< 0.001	6.62	-6	14	50	
***Insula/Striatal circuitry***		0.020	244						
Insula lobe	R			0.010	5.19	39	14	8	
Caudate nucleus	R			0.105	4.31	21	14	5	
Putamen	R			0.226	3.88	24	5	11	
***Lateral PFC***		0.025	209						
Middle Frontal gyrus	L			0.019	4.94	-39	32	35	
Middle Frontal gyrus	L			0.042	4.66	-39	38	29	
***SPL/Postcentral gyrus***	R	0.020	271	0.001	5.89	36	-43	62	
***SPL/Postcentral gyrus***		0.027	190						
SPL	L			0.014	5.07	-30	-55	68	
SPL	L			0.226	3.90	-45	-43	62	
Inf. Parietal lobule	L			0.321	3.73	-39	-37	47	

*Note* Results were FDR cluster corrected (*p*_*FDRcc*_<0.05) with a primary voxel-wise threshold of *p* <.001. Peak coordinates of 5 local maxima more than 4 mm apart are reported. Dotted lines represent different clusters of activation. R/L = Right or left hemisphere, K = voxels in the cluster, Sup. = superior, Inf. = inferior, SMA = Supplementary Motor Area, PFC = Prefrontal Cortex, SPL = Superior Parietal Lobule

The reverse contrast (Inclusion Ball vs. Exclusion No Ball) led to five clusters with increased activity, including the insula/stiatal circuitry, lateral PFC and superior motor area (SMA; see Fig. 3B; Table 2).

**Fig. 4:**
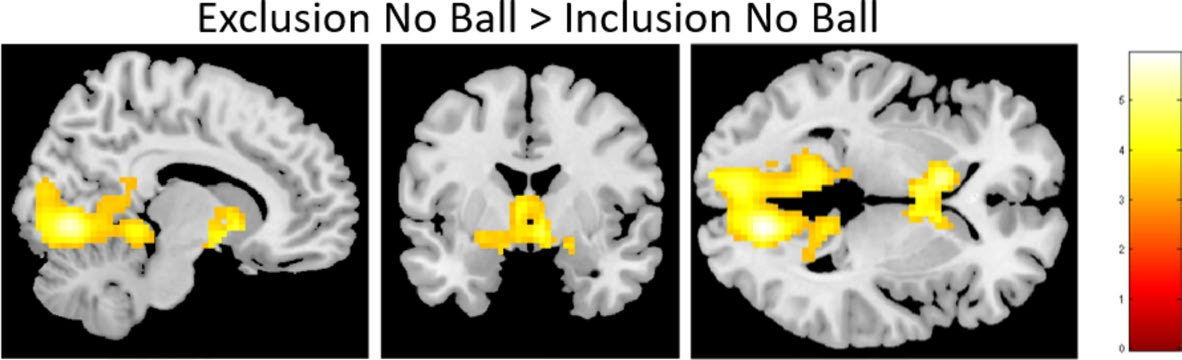
Brain activity during explicit vs. incidental exclusion in the contrast Exclusion No Ball vs. Inclusion No Ball

**Exclusion No Ball vs. Inclusion No Ball.** Next, we examined neural correlates of social exclusion with the whole-brain *t*-contrast of not receiving the ball in the exclusion vs. not receiving the ball in the inclusion block. This contrast resulted in two clusters of activation in the occipital lobe and the ventral striatum (see Fig. 4; Table 3). The reverse contrast did not result in significant clusters of activation.

**Table 3:** Activity in whole brain t-contrast ‘Exclusion No Ball vs. inclusion no ball’

Brain region	*R*/L	Cluster-level			Peak-level				
		*p* (FDR)	K		*p* (FDR)	*t*	MNI coordinates		
							x	y	z
Exclusion No Ball > Inclusion No Ball									
***Occipital lobe***		< 0.001	1877						
Lingual gyrus	R				0.010	5.95	12	-73	-4
Lingual gyrus	L				0.061	5.10	-9	-85	-7
Lingual gyrus	L				0.123	4.65	-6	-73	-10
Calcarine gyrus	L				0.150	4.43	-9	-97	2
Lingual gyrus	L				0.150	4.42	-15	-49	-7
***Striatal circuitry***		0.002	519						
Putamen	L				0.033	5.40	-12	8	-7
Caudate nucleus	L				0.090	4.89	-6	2	2
Caudate nucleus	R				0.123	4.64	6	-1	-7
Amygdala	R				0.123	4.62	18	5	-10
Inf. Frontal gyrus	L				0.150	4.50	-36	29	-13

*Note* Results were FDR cluster corrected (*p*_*FDRcc*_<0.05) with a primary voxel-wise threshold of *p* <.001. Peak coordinates of 5 local maxima more than 4 mm apart are reported. Dotted lines represent different clusters of activation. R/L = Right or left hemisphere, K = voxels in the cluster, Inf. = Inferior

**Fig. 5:**
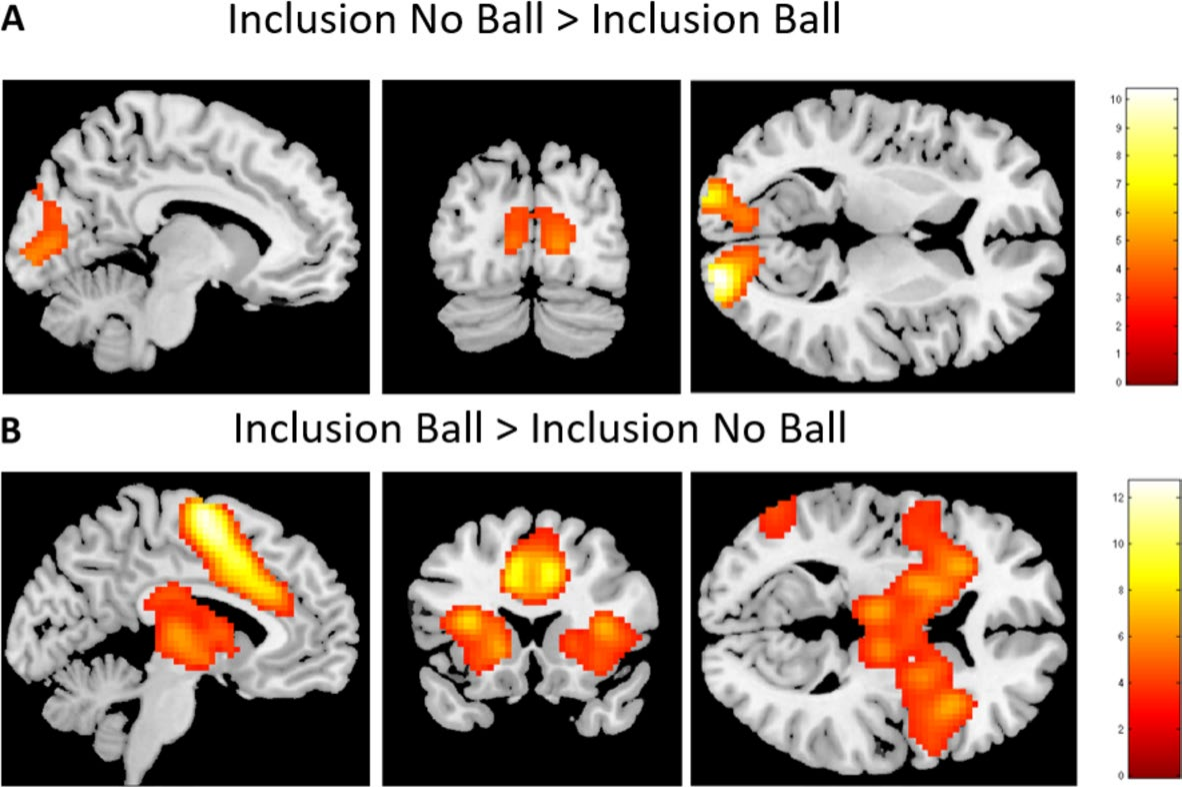
Brain activity during incidental exclusion, and reversed contrast. **A**) contrast Inclusion No Ball vs. Inclusion Ball. **B**) contrast Inclusion Ball vs. Inclusion No Ball

**Inclusion No Ball vs. Inclusion Ball.** We finally examined neural correlates of incidental exclusion by examining the whole brain *t*-contrast of not receiving the ball in the inclusion block vs. receiving the ball in the inclusion block. This analysis resulted in activation only in the occipital lobe (see Table 4). The reverse contrast (Inclusion Ball vs. Inclusion No Ball) resulted in a wide network of activation, including the insula, lateral PFC and SMA (see Fig. 5, and Table 4).

**Table 4:** Activity in whole brain t-contrasts ‘inclusion no ball vs. inclusion ball’ and ‘inclusion ball vs. inclusion no ball’

Brain region	*R*/L	Cluster-level			Peak-level									
		*p* (FDR)	K		*p* (FDR)		*t*		MNI coordinates					
									x		y		z	
Incidental exclusion: Inclusion No Ball > Inclusion Ball														
***Occipital Lobe***		< 0.001	929											
Sup. Occipital gyrus	R			< 0.001		10.32		21		-94		5		
Middle Occipital gyrus	L			< 0.001		7.68		-21		-97		5		
Calcarine gyrus	R			0.296		3.87		9		-76		17		
Cuneus	L			0.300		3.72		-9		-82		20		
Inclusion Ball > Inclusion No Ball														
***SMA***		< 0.001	3396											
SMA	L			< 0.001		12.70		-9		-4		65		
SMA	R			< 0.001		11.17		9		2		56		
SMA	L			< 0.001		9.84		-6		11		44		
SMA	R			< 0.001		9.65		12		-4		68		
Middle cingulate cortex	R			< 0.001		9.36		9		14		38		
***Insula/striatal circuitry***		< 0.001	3230											
Putamen	L			< 0.001		8.97		-15		8		-4		
Insula lobe	L			< 0.001		7.77		-33		17		14		
Putamen	R			< 0.001		6.84		24		8		2		
Insula lobe	R			< 0.001		6.82		36		17		11		
Thalamus	L			< 0.001		5.91		-9		16		8		
***SPL/postcentral***		0.017	376											
Postcentral gyrus	R			0.001		5.82		33		-43		68		
Postcentral gyrus	R			0.009		5.18		39		-37		59		
Supramarginal gyrus	R			0.325		3.75		63		-37		44		
Supramarginal gyrus	R			0.553		3.49		66		-43		38		
***Occipital lobe***		0.020	332											
Middle Occipital gyrus	L			0.027				-54		-70		-1		
Middle Occipital gyrus	L			0.028				-57		-67		2		
Inf. Occipital gyrus	L			0.073				-42		-70		-7		

*Note* Results were FDR cluster corrected (*p*_*FDRcc*_<0.05) with a primary voxel-wise threshold of *p* <.001. Peak coordinates of 5 local maxima more than 4 mm apart are reported. Dotted lines represent different clusters of activation. R/L = Right or left hemisphere, K = voxels in the cluster, SMA = Supplementary Motor Area, SPL = Superior Parietal Lobule, Inf. = Inferior

### ROI Analyses

In ROI analyses, we checked whether activation in the insula, dACC, IFG and lateral PFC during social exclusion was related to victimization, using separate multivariate tests for repeated and recent victimization.

**Exclusion No Ball vs. Inclusion Ball.** Results of the multivariate tests showed that ROI activation was not predicted by either repeated victimization (*F*(4,68) = 1.27, *p* =.290, *η*^*2*^_*p*_ = 0.07) or recent victimization (*F*(4,70) = 1.70, *p* =.161, *η*^*2*^_*p*_ = 0.09; Table 5). Univariate results showed that recent victimization predicted activation in the bilateral insula (*β* = 0.27, *F*(1,73) = 5.33, *p* =.024, *η*^*2*^_*p*_ = 0.07), such that higher victimization scores were associated with increased insula activation during explicit exclusion vs. inclusion (Supplementary Figure S1a).

**Table 5:** Statistics of the ROI analyses on the relation between neural activity and repeated victimization, neural activity and recent victimization, and neural activity and intention to punish, using independent ROIs (from the automated anatomical labeling atlas, Will et al., 2016a, b; Rudolph et al. (2016); Asscheman et al. (2019)

	Repeated victimization				Recent victimization				Intention to punish			
	*F*	*df*	*p*	*η* ^*2*^_*p*_	*F*	*df*	*p*	*η* ^*2*^_*p*_	*F*	*df*	*p*	*η* ^*2*^_*p*_
Exclusion No Ball > Inclusion Ball												
Multivariate test	1.27	4,68	.290	.07	1.70	4,70	.161	.09	1.33	4,70	.268	.07
Insula	3.35	1,71	.072	.04	5.33	1,73	.024*	.07	2.33	1,73	.131	.03
dACC	0.49	1,71	.488	.01	0.19	1,73	.665	<.01	0.05	1,73	.829	<.01
IFG	0.01	1,71	.944	<.01	0.47	1,73	.497	.01	0.06	1,73	.802	<.01
dlPFC	0.45	1,71	.504	.01	0.38	1,73	.540	.01	1.37	1,73	.245	.02
Exclusion No Ball > Inclusion No Ball												
Multivariate test	0.93	4,68	.454	.05	1.68	4,70	.165	.09	2.37	4,70	.060	.12
Insula	2.90	1,71	.093	.04	4.47	1,73	.038*	.06	7.40	1,73	.008**	.09
dACC	0.01	1,71	.944	<.01	0.46	1,73	.502	.01	0.00	1,73	1.00	<.01
IFG	0.22	1,71	.644	<.01	1.45	1,73	.232	.02	0.08	1,73	.780	<.01
dlPFC	0.06	1,71	.814	<.01	0.11	1,73	.724	<.01	0.64	1,73	.426	.01
Inclusion No Ball > Inclusion Ball												
Multivariate test	0.31	4,68	.867	.02	0.73	4,70	.578	.04	1.34	4,70	.262	.07
Insula	0.05	1,71	.816	<.01	0.14	1,73	.710	<.01	2.06	1,73	.155	.03
dACC	0.70	1,71	.407	.01	0.26	1,73	.609	<.01	0.08	1,73	.780	<.01
IFG	0.16	1,71	.690	<.01	1.02	1,73	.317	.01	0.02	1,73	.904	<.01
dlPFC	0.13	1,71	.721	<.01	1.12	1,73	.293	.02	0.21	1,73	.651	<.01

*Note* *p <.05, **p <.01. Abbreviations: dACC = dorsal anterior cingulate cortex; IFG = inferior frontal gyrus; dlPFC = dorsolateral prefrontal cortex

**Exclusion No Ball vs. Inclusion No Ball.** Multivariate results showed that ROI activation was not predicted by repeated victimization (*F*(4,68) = 0.93, *p* =.454, *η*^*2*^_*p*_ = 0.05), and not by recent victimization (*F*(4,70) = 1.68, *p* =.165, *η*^*2*^_*p*_ = 0.09; Table 5). Again, in univariate tests, recent victimization was predictive of activation in the bilateral insula (*β* = 0.25, *F*(1,73) = 4.47, *p* =.038, *η*^*2*^_*p*_ = 0.06), such that higher victimization scores were associated with increased insula activation during explicit exclusion vs. incidental exclusion (Supplementary Figure S1b).

**Inclusion No Ball vs. Inclusion Ball.** Multivariate tests revealed that ROI activation was not predicted by repeated victimization (*F*(4,68) = 0.31, *p* =.867, *η*^*2*^_*p*_ = 0.02), and not by recent victimization (*F*(4,70) = 0.73, *p* =.578, *η*^*2*^_*p*_ = 0.04; Table 5).

#### Behavioral Results

**Fig. 6:**
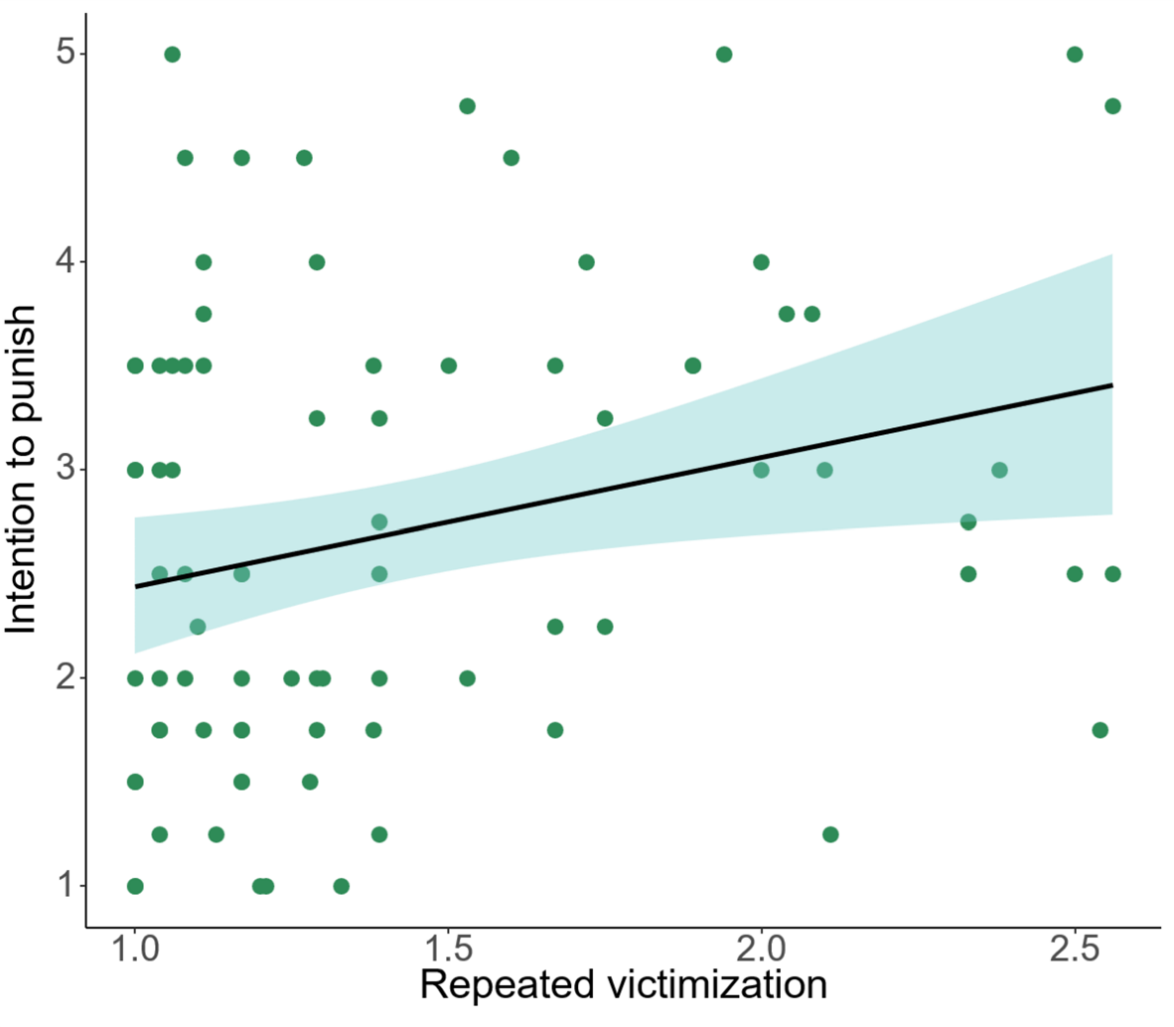
Intention to punish correlated significantly with repeated victimization. Adjusted outliers are used, repeated victimization accounted for 6.7% of variance

Regressing intention to punish on repeated victimization proved significant (*β* = 0.26, *F*(1,80) = 5.74, *p* =.019, *η*^*2*^_*p*_ = 0.07, see Fig. 6). Individuals that experienced higher levels of victimization intensity over the past two years, were more inclined to punish the excluders in the Cyberball game. In contrast, recent victimization did not relate to intention to punish (*β* = 0.15, *F*(1,82) = 1.94, *p* =.168, *η*^*2*^_*p*_ = 0.02).

### Explorative Analyses

#### Brain Activity and Intention to Punish

Finally, we explored whether brain activity in the four ROIs in the three contrasts of interest was related to intentions to punish. None of the multivariate regressions were significant (all *F*’s ≤ 2.37, *p*’s ≥ 0.060; see Table 5). However, in univariate regressions, intention to punish was predicted by bilateral insula activation in the *Exclusion No Ball > Inclusion No Ball* contrast (*β* = 0.31, *F*(1,73) = 7.40, *p* =.008, *η*^*2*^_*p*_ = 0.09), such that increased insula activation was associated to increased intention to punish (Supplementary Figure S2).

## Discussion

The overarching aim of this study was to examine how repeated experiences of victimization in 8- to 12-year-olds relate to neural processing of and behavioral responses to social exclusion. Based on the literature, we hypothesized that victimization experiences would relate to stronger responses in the insula, ACC and lPFC during social exclusion (vs. inclusion). Based on the SIP model, we hypothesized that victimization experiences would positively correlate with retaliation goals as reflected by intentions to punish excluders. Repeated and recent victimization were not related to neural processing of social exclusion, although there were some indications that insula activity during social exclusion (vs. inclusion) correlated positively with recent victimization experiences. Similarly, neural processing of social exclusion was not related to intentions to punish, although there was some evidence that insula activity during social inclusion (vs. exclusion) related negatively to intentions to punish. Finally, we showed that only repeated (and not recent) victimization experiences related to increased intentions to punish excluders.

### Peer Victimization and Behavioral Responses to Social Exclusion

We found that children generally experience exclusion as aversive, as reflected in lower mood and need satisfaction after social exclusion elicited through the Cyberball paradigm. However, only repeated victimization related positively to intentions to punish excluders. Generally, all children are inclined to punish excluders (Gunther Moor et al., 2012; Will et al., 2015; Will, Crone, et al., 2016a; Will et al., 2016b), yet in previous research peer adversity was not related to increased punishment intentions (Will, Crone, et al., 2016a; Will et al., 2016b). Possibly, more intense experiences like victimization (compared to peer rejection) drive the association. Another possibility is that the intention to punish may be driven by need satisfaction. Everyone feels worse after being excluded, however, children who feel less satisfied during inclusion (and after debriefing) were more inclined to punish excluders. Hence, increased intentions to punish others might depend more on their general state (general feelings of belonging) than on specific incidents. This interpretation would be in line with our finding that repeated victimization related positively to intentions to punish excluders, yet recent victimization did not. To further substantiate these results, future research could include intentions to punish includers and neutral others (Gunther Moor et al., 2012) and relate this to victimization experiences. This way it can be examined whether the finding of repeated victimization and intention to punish generalizes over different types of peers involved in interactions. Furthermore, as first impressions typically last (Lee et al., 2016), excluders could make a comeback in a follow-up game where they act as includers, to see whether the intentions to punishment last when peers adjust their behavior. With these suggested extensions to research, the social intricacies in relation to (repeated) victimization experiences can be further unraveled.

### Neural Responses to Social Exclusion

In examining the neural correlates of social exclusion, we focused both on explicit exclusion (i.e., not receiving a ball in the exclusion block vs. receiving a ball in the inclusion block) and on incidental exclusion (i.e., not receiving a ball in the inclusion block vs. receiving a ball in the inclusion block). We found indications that explicit social exclusion related to heightened neural responses in the (bilateral) paracentral lobule and the (bilateral) occipital lobe, which is only partly in line with prior research (Mwilambwe-Tshilobo & Spreng, 2021; Vijayakumar et al., 2017). Contrary to our expectations, explicit social exclusion did not result in neural activation in the insula or lateral PFC, although uncorrected whole-brain analyses did show activation in these regions (untresholded statistical maps of the whole-brain contrasts are available on Neurovault (https://identifiers.org/neurovault.collection:18019)). Another prior finding that was not replicated in the current study is the involvement of ACC during social exclusion (e.g., Cheng et al., 2020; Will et al., 2016). Possibly this is due to the few severe chronic victimized children in our sample, as Will, van Lier and colleagues compared severe long-term rejected over six years vs. stably accepted adolescents. Alternatively, the explanation may lie in the examined age groups. The children in our study were younger than in, for example, Will, van Lier and colleagues’ study, and ACC activity during exclusion has been found predominantly in (young) adult samples and not in developmental samples (Vijayakumar et al., 2017), highlighting the importance of studies into (young) developmental samples.

Similarly, when we examined neural processing of incidental exclusion (i.e., not receiving a ball in the inclusion block vs. receiving a ball in the inclusion block), only an occipital cluster showed heightened activity. Finding an activated occipital cluster was not unique, as there was also an activated occipital cluster during explicit exclusion, and likely reflects visual attention to the stimulus (moving ball and figures). Previous findings for this contrast have been inconsistent, as a study with participants in the same age range as the current study found no heightened activity clusters (Asscheman et al., 2019); yet two other studies with adolescents found similar activation clusters as during explicit exclusion (de Water et al., 2017; Will et al., 2016b). The studies with activated clusters had participants that were in the (12–17) adolescent age range, so perhaps they were at their prime for social sensitivity. In contrast, the participants in our study were still in (late) childhood (8–12 years) and may not have been as socially sensitive yet. Replication of these specific contrasts (incidental and explicit exclusion) in different (age) samples is needed to determine whether incidental exclusion is processed more similarly to explicit exclusion, to understand how the brain processes incidental instances of exclusion over the course of late childhood to adolescence.

We also examined explicit exclusion vs. incidental exclusion (i.e., not receiving a ball in the exclusion block vs. not receiving a ball in the inclusion block), which is very rarely examined in the literature. In line with previous research, we found that explicit exclusion was associated with increased activity in the occipital and striatal regions (Will et al., 2016), indicating that explicit exclusion is *not* processed the same as incidental exclusion. Other studies have also shown activity in parts of the cingulate cortex (Asscheman et al., 2019; Schulz et al., 2022). The striatal region has been shown to play an important role in (social) learning and prediction errors (Báez-Mendoza & Schultz, 2013; Schönberg et al., 2007). Moreover, the activated striatal circuitry also included the amygdala and IFG. The IFG has been associated with processing unpleasantness (negative vs. neutral stimuli) (Sambuco et al., 2020), and has been implicated in cognitive control. This suggests that the negative social experiences during the exclusion block (i.e., explicit exclusion) might be more salient and possibly requires more cognitive control than the (negative) experiences during the inclusion block (i.e., incidental exclusion).

Finally, we examined neural correlates of being included (i.e., receiving balls in the inclusion block vs. not receiving balls in the exclusion block). These contrasts resulted in widespread neural activation including the (right) insula/striatal circuitry, (left) lateral PFC and bilateral SMA, in line with prior findings showing that inclusion experiences involve (pre)motor activity and reward processes (Gunther Moor et al., 2012; Puetz et al., 2014; Schulz et al., 2022). Whereas the anterior insula has been commonly found more activated by negative stimuli (Büchel et al., 1998; Carretié et al., 2009; Mériau et al., 2009), the middle insula has been found to be increasingly activated by more pleasant stimuli (Bartels & Zeki, 2004). A previous study found that putamen and insula activity could act as tracers of social cue accuracy to receive rewards (Henco et al., 2020). Another study found insula/striatal activity when receiving positive feedback from peers, especially for very low or very high levels of adversity (Rudolph et al., 2021). Together with these previous findings, our results suggest that the insula/striatal activation during inclusion may be related to experiencing a pleasant social interaction. Our inclusion ball vs. incidental exclusion contrast led to similar activity clusters, which strengthens this interpretation.

### Neural Responses to Social Exclusion, Peer Victimization and Intention to Punish

In ROI analyses, we examined whether children with victimization experiences showed differential neural processing in regions previously associated with social exclusion, i.e., the insula, dACC, IFG and dlPFC. Repeated and recent victimization were not related to neural processing of explicit exclusion or incidental exclusion in these regions, which may be partly explained by the relatively low variability in victimization scores in the sample. However, while it did not survive multiple comparison corrections, the univariate results gave some indications that recent victimization related positively to activity in the insula during explicit exclusion (both relative to inclusion and incidental exclusion), which is in line with previous research on victimization and social exclusion (Kiefer et al., 2021; McIver et al., 2018). Given that the insula has been related to threat processing and cognitive control functions (Puiu et al., 2020; Sambuco et al., 2020; Tops & Boksem, 2011), it is possible that being socially excluded is a more intense experience for children who are victimized, requiring more cognitive control. This interpretation was paralleled by our behavioral findings that children with higher victimization scores had more intentions to punish excluders. Importantly, the interactions in our study were with unfamiliar peers. Therefore, interactions with personally familiar peers, such as classmates (and bullies), are likely to elicit even stronger responses. Currently, neuroscientific research on exclusion by familiar peers is lacking. It is very difficult to include personally familiar peers in experimental paradigms, especially those with negative relationships such as disliked peers and bullies (see Güroǧlu & Veenstra, 2021 for a discussion). Nevertheless, given that the effects did not survive multiple comparison corrections and had small to medium effect sizes (Cohen, 1969; Richardson, 2011), future research should replicate our findings and also aim to examine the influence of familiarity with peers as bullying is often done by familiar peers.

In exploratory analyses, we tested whether neural responses during social exclusion were related to intention to punish excluders in the Cyberball game. Results did not reveal associations between neural processing across ROIs and intention to punish. However, univariate tests of the separate ROIs provided some indications that increased activity in the insula during explicit exclusion (vs. incidental exclusion) was related to increased intention to punish excluders. This finding is in line with prior studies showing that insula activation was related to increased aggression in late childhood (Achterberg et al., 2020), and decreased prosocial behaviors in childhood and adulthood (Schreuders et al., 2018; van der Meulen et al., 2018).

## Strengths, Limitations, and Conclusion

This study is among the first to examine associations between neural processing of social exclusion and repeated victimization experiences in childhood. A main strength of this study was the recruitment of children with available victimization data over the past two years. The reports were collected prospectively instead of retrospectively, hence, effects of memory and current experiences were reduced in the victimization measures. Furthermore, we had a substantially large sample, which improves the reliability of the results compared with previous studies. We examined uncommon contrasts, all related to SIP theory, to examine neural processing of incidental exclusion versus explicit exclusion. Last, we are among the first studies to combine different phases of the SIP model, interpretation and response, in combination with neuroscientific measures to better examine the whole SIP cycle.

Our study also had some limitations. The recruitment and data collection took part during the corona pandemic, because of which we were not able to recruit face-to-face at schools and had to recruit through e-mail and videos. This likely resulted in the higher SES levels in our sample. Furthermore, coming approximately three hours to the hospital during the pandemic also requires more involved or supportive parents. As persistent victims typically have lower quality parent-child relationships (Kaufman et al., 2018), the required involvement and effort of parents in the current study might have led to a lower rate of children experiencing persistent victimization. The likely positive home environment, support and higher SES, might buffer aggressive responses to social exclusion. Our conclusions are therefore limited to milder victimization instead of severe victimization, and we emphasize the need for replication in samples with more severe rates of victimization. Moreover, as data was collected in The Netherlands, future studies may explore the results in light of possible cultural differences. In addition, victimization was defined as being the receiver of repeated, intentionally aggressive or hurtful behavior, that might be in the form of being excluded or rejected but also in the form of being exposed to explicit, direct or physical aggressive behaviors. Depending on the type of victimization (e.g., relational, physical or verbal victimization), participants may possibly have differed in their response to social exclusion. Future studies with more heterogeneity in victimization could help disentangle the effects of social exclusion for different forms of victimization. Finally, the inclusion and exclusion trials in the Cyberball game were divided into separate blocks, as is common in traditional Cyberball designs (Mwilambwe-Tshilobo & Spreng, 2021). However, because the blocks were presented during separate fMRI runs, it is difficult to disentangle task effects from effects of restarting the scanner. Future studies may use alternating Cyberball designs to overcome this.

Overall, this study focused on the neural processing of social exclusion and how it relates to repeated victimization in 8–12-year old children. Victimization did not relate to neural activation during social exclusion, although there were indications that recent victimization may relate to increased insula activation during explicit exclusion (but not incidental exclusion). Furthermore, repeated victimization related to more intentions to punish excluders. Neural activation during social exclusion across ROIs did not predict intention to punish others, but results tentatively suggested that increased insula activation during social exclusion may relate to increased intention to punish. These findings seem to suggest that children with victimization experiences may have more difficulty dealing with social exclusion experiences, even when these peers were previously unknown. This could be used in interventions to educate peers, teachers, and parents to increase compassion and understanding, also when children who are victimized respond strongly, to reduce subsequent rejection of victimized children. Interventions could also benefit from strengthening behavioral control of children to decrease experiences of victimization. Future research should substantiate these results. For now, children who are victimized seem to experience and respond to social exclusion more strongly, which should be kept in mind by all that are involved.

## Electronic Supplementary Material

Below is the link to the electronic supplementary material.

Supplementary Material 1

## Data Availability

The larger project (https://osf.io/vnxtq) as well as the current study (https://osf.io/3fhku) have been pre-registered; see https://osf.io/feg3r for deviations from the pre-registration. Data, matlab scripts and SPSS syntax are available by contacting the corresponding author. Untresholded statistical maps of the whole-brain contrasts are available on Neurovault (https://identifiers.org/neurovault.collection:18019).

## References

[CR1] Achterberg, M., Van Duijvenvoorde, A. C., van IJzendoorn, M. H., Bakermans-Kranenburg, M. J., & Crone, E. A. (2020). Longitudinal changes in DLPFC activation during childhood are related to decreased aggression following social rejection. *Proceedings of the National Academy of Sciences*, *117*(15), 8602–8610. 10.1073/pnas.1915124117. 10.1073/pnas.1915124117 PMC7165424 32234781

[CR2] Asscheman, J. S., Koot, S., Ma, I., Buil, J. M., Krabbendam, L., Cillessen, A. H. N., & van Lier, P. A. C. (2019). Heightened neural sensitivity to social exclusion in boys with a history of low peer preference during primary school. *Developmental Cognitive Neuroscience*, *38*(2019), 100673. 10.1016/j.dcn.2019.100673. 31252200 10.1016/j.dcn.2019.100673 PMC6969346

[CR3] Báez-Mendoza, R., & Schultz, W. (2013). The role of the striatum in social behavior. *Frontiers in Neuroscience*, *7*(7 DEC), 1–14. 10.3389/fnins.2013.00233. 10.3389/fnins.2013.00233 PMC3857563 24339801

[CR4] Bartels, A., & Zeki, S. (2004). The neural correlates of maternal and romantic love. *Neuroimage*, *21*(3), 1155–1166. 10.1016/j.neuroimage.2003.11.003. 15006682 10.1016/j.neuroimage.2003.11.003

[CR5] Baumeister, R. F., & Leary, M. R. (1995). The need to belong: Desire for interpersonal attachments as a fundamental human motivation. *Psychological Bulletin*, *117*(3), 497–529. 10.1037/0033-2909.117.3.497. 7777651

[CR6] Bowes, L., Maughan, B., Ball, H., Shakoor, S., Ouellet-Morin, I., Caspi, A., & Arseneault, L. (2013). Chronic bullying victimization across school transitions: The role of genetic and environmental influences. *Development and Psychopathology*, *25*(2), 333–346. 10.1017/S0954579412001095. 23627948 10.1017/S0954579412001095 PMC3881278

[CR8] Büchel, C., Morris, J., Dolan, R. J., & Friston, K. J. (1998). Brain systems mediating aversive conditioning: An event-related fMRI study. *Neuron*, *20*(5), 947–957. 10.1016/S0896-6273(00)80476-6. 9620699 10.1016/s0896-6273(00)80476-6

[CR9] Calvete, E., Fernández-González, L., González-Cabrera, J. M., & Gámez-Guadix, M. (2018). Continued bullying victimization in adolescents: Maladaptive schemas as a mediational mechanism. *Journal of Youth and Adolescence*, *47*(3), 650–660. 10.1007/s10964-017-0677-5. 28434091 10.1007/s10964-017-0677-5

[CR10] Camodeca, M., & Goossens, F. A. (2005). Aggression, social cognitions, anger and sadness in bullies and victims. *Journal of Child Psychology and Psychiatry and Allied Disciplines*, *46*(2), 186–197. 10.1111/j.1469-7610.2004.00347.x. 15679527 10.1111/j.1469-7610.2004.00347.x

[CR11] Carretié, L., Albert, J., López-Martín, S., & Tapia, M. (2009). Negative brain: An integrative review on the neural processes activated by unpleasant stimuli. *International Journal of Psychophysiology*, *71*, 57–63. 18727941 10.1016/j.ijpsycho.2008.07.006

[CR12] Cheng, T. W., Vijayakumar, N., Flournoy, J. C., op de Macks, Z., Peake, S. J., Flannery, J. E., Mobasser, A., Alberti, S. L., Fisher, P. A., & Pfeifer, J. H. (2020). Feeling left out or just surprised? Neural correlates of social exclusion and overinclusion in adolescence. *Cognitive Affective and Behavioral Neuroscience*, *20*(2), 340–355. 10.3758/s13415-020-00772-x. 10.3758/s13415-020-00772-x PMC7338003 32056138

[CR15] Cohen, J. (1969). *Statistical power analysis for the behavioural sciences*. Academic.

[CR16] Crick, N. R., & Dodge, K. A. (1994). Review and reformulation of social information-processing mechanisms in children’s social adjustment. *Psychological Bulletin*, *115*(1), 74–101. 10.1037/0033-2909.115.1.74.

[CR17] de Water, E., Mies, G. W., Ma, I., Mennes, M., Cillessen, A. H. N., & Scheres, A. (2017). Neural responses to social exclusion in adolescents: Effects of peer status. *Cortex; a Journal Devoted to the Study of the Nervous System and Behavior*, *92*, 32–43. 10.1016/j.cortex.2017.02.018. 28395165 10.1016/j.cortex.2017.02.018

[CR18] Eisenberger, N. I., Lieberman, M. D., & Williams, K. D. (2003). Does rejection hurt- An fMRI study of social exclusion. *Science*, *302*, 290–292. 14551436 10.1126/science.1089134

[CR20] Gunther Moor, B., Güroǧlu, B., de Macks, O., Rombouts, Z. A., van der Molen, S. A. R. B., M. W., & Crone, E. A. (2012). Social exclusion and punishment of excluders: Neural correlates and developmental trajectories. *Neuroimage*, *59*(1), 708–717. 10.1016/j.neuroimage.2011.07.028. 21791248 10.1016/j.neuroimage.2011.07.028

[CR21] Güroǧlu, B., & Veenstra, R. (2021). Neural underpinnings of peer experiences and interactions: A review of social neuroscience. *Merrill-Palmer Quarterly*, *67*(4), 416–456. https://www.muse.jhu.edu/article/856076.

[CR22] Haltigan, J. D., & Vaillancourt, T. (2014). Joint trajectories of bullying and peer victimization across elementary and middle school and associations with symptoms of psychopathology. *Developmental Psychology*, *50*(11), 2426. 10.1037/a0038030. 25313592 10.1037/a0038030

[CR23] Hartgerink, C. H. J., Van Beest, I., Wicherts, J. M., & Williams, K. D. (2015). The ordinal effects of ostracism: A meta-analysis of 120 cyberball studies. *Plos One*, *10*(5), e0127002. 10.1371/journal.pone.0127002. 26023925 10.1371/journal.pone.0127002 PMC4449005

[CR24] Hawker, D. S. J., & Boulton, M. J. (2000). Twenty years’ research on peer victimization and psychosocial maladjustment: A meta-analytic review of cross-sectional studies. *Journal of Child Psychology and Psychiatry and Allied Disciplines (Vol*, *41*(4), 441–455. 10.1017/S0021963099005545. 10836674

[CR25] Henco, L., Brandi, M. L., Lahnakoski, J. M., Diaconescu, A. O., Mathys, C., & Schilbach, L. (2020). Bayesian modelling captures inter-individual differences in social belief computations in the putamen and insula. *Cortex; a Journal Devoted to the Study of the Nervous System and Behavior*, *131*, 221–236. 10.1016/j.cortex.2020.02.024. 32571519 10.1016/j.cortex.2020.02.024

[CR26] Kaufman, T. M. L., Kretschmer, T., Huitsing, G., & Veenstra, R. (2018). Why does a universal anti-bullying program not help all children? Explaining persistent victimization during an intervention. *Prevention Science*, *19*(6), 822–832. 10.1007/s11121-018-0906-5. 29707731 10.1007/s11121-018-0906-5

[CR28] Kellij, S., Lodder, G., van den Bedem, N., Güroğlu, B., & Veenstra, R. (2022). The social cognitions of vicitms of bullying: A systematic review. *Adolescent Research Review*, *7*, 287–334. 10.1007/s40894-022-00183-8.

[CR27] Kellij, S., Lodder, G. M. A., Giletta, M., Zimmer-Gembeck, M. J., Güroǧlu, B., & Veenstra, R. (2023). Are there negative cycles of peer victimization and rejection sensitivity? Testing ri-CLPMs in two longitudinal samples of young adolescents. *Development and Psychopathology*, 1–13. 10.1017/S0954579423000123. 10.1017/S0954579423000123 PMC7616006 36794389

[CR29] Kiefer, M., Sim, E. J., Heil, S., Brown, R., Herrnberger, B., Spitzer, M., & Grön, G. (2021). Neural signatures of bullying experience and social rejection in teenagers. *Plos One*, *16*(8 August), 1–16. 10.1371/journal.pone.0255681. 10.1371/journal.pone.0255681 PMC8341587 34351976

[CR30] Lansu, T. A. M., van Noorden, T. H. J., & Deutz, M. H. F. (2017). How children’s victimization relates to distorted versus sensitive social cognition: Perception, mood, and need fulfillment in response to cyberball inclusion and exclusion. *Journal of Experimental Child Psychology*, *154*, 131–145. 10.1016/j.jecp.2016.10.012. 27875750 10.1016/j.jecp.2016.10.012

[CR31] Lee, N. C., Jolles, J., & Krabbendam, L. (2016). Social information influences trust behavior in adolescents. *Journal of Adolescence*, *46*, 66–75. 10.1016/j.adolescence.2015.10.021. 26599529 10.1016/j.adolescence.2015.10.021

[CR32] Mazzone, A., Yanagida, T., Camodeca, M., & Strohmeier, D. (2021). Information processing of social exclusion: Links with bullying, moral disengagement and guilt. *Journal of Applied Developmental Psychology*, *75*, 101292. 10.1016/j.appdev.2021.101292.

[CR33] McDonald, K. L., & Asher, Steven, R. (2018). Peer acceptance, peer rejection, and popularity. In W. M. Bukowski, B. Laursen, & K. H. Rubin (Eds.), *Handbook of peer interactions, relationships, and groups* (2nd ed., pp. 429–446). The Guilford Press.

[CR34] McIver, T. A., Bosma, R. L., Sandre, A., Goegan, S., Klassen, J. A., Chiarella, J., Booij, L., & Craig, W. (2018). Peer victimization is associated with neural response to social exclusion. *Merrill-Palmer Quarterly*, *64*(1), 135–161. 10.13110/merrpalmquar1982.64.1.0135.

[CR35] Mellin, E. A. (2012). Relational victimization and rejection sensitivity: The long-term impact of social hurt. *Adultspan Journal*, *11*(1), 2–15. 10.1002/j.2161-0029.2012.00001.x.

[CR36] Mériau, K., Wartenburger, I., Kazzer, P., Prehn, K., Villringer, A., van der Meer, E., & Heekeren, H. R. (2009). Insular activity during passive viewing of aversive stimuli reflects individual differences in state negative affect. *Brain and Cognition*, *69*, 73–80. 18632198 10.1016/j.bandc.2008.05.006

[CR37] Mwilambwe-Tshilobo, L., & Spreng, R. N. (2021). Social exclusion reliably engages the default network: A meta-analysis of Cyberball. *Neuroimage*, *227*. 10.1016/j.neuroimage.2020.117666. (May 2020. 10.1016/j.neuroimage.2020.117666 PMC8005222 33359341

[CR38] Olweus, D. (1996). Revised Olweus bully/victim questionnaire. *Journal of Psychopathology and Behavioral Assessment*. 10.1037/t09634-000.

[CR39] Park, A., Jensen-Campbell, L. A., & Miller, H. L. (2017). The effects of peer relational victimization on social cognition: Rejection attribution bias or a more generalized sensitivity to social pain? *Journal of Social and Personal Relationships*, *34*(7), 984–1006. 10.1177/0265407516664418.

[CR40] Pharo, H., Gross, J., Richardson, R., & Hayne, H. (2011). Age-related changes in the effect of ostracism. *Social Influence*, *6*(1), 22–38. 10.1080/15534510.2010.525852.

[CR41] Puetz, V. B., Kohn, N., Dahmen, B., Zvyagintsev, M., Schultz, R. T., Heim, C. M., Fink, G. R., Herpertz-Dahlmann, B., & Konrad, K. (2014). Neural response to social rejection in children with early separation experiences. In *Journal of the American Academy of Child and Adolescent Psychology* (Vol. 53, Issue 12). www.jaacap.org. 10.1016/j.jaac.2014.09.004 25457931

[CR42] Puiu, A. A., Wudarczyk, O., Kohls, G., Bzdok, D., Herpertz-Dahlmann, B., & Konrad, K. (2020). Meta-analytic evidence for a joint neural mechanism underlying response inhibition and state anger. *Human Brain Mapping*, *41*(11), 3147–3160. 10.1002/hbm.25004. 32314475 10.1002/hbm.25004 PMC7336147

[CR44] Reijntjes, A., Kamphuis, J. H., Prinzie, P., & Telch, M. J. (2010). Peer victimization and internalizing problems in children: A meta-analysis of longitudinal studies. *Child Abuse and Neglect*, *34*(4), 244–252. 10.1016/j.chiabu.2009.07.009. 20304490 10.1016/j.chiabu.2009.07.009

[CR43] Reijntjes, A., Kamphuis, J. H., Prinzie, P., Boelen, P. A., van der Schoot, M., & Telch, M. J. (2011). Prospective linkages between peer victimization and externalizing problems in children: A meta-analysis. *Aggressive Behavior*, *37*(3), 215–222. 10.1002/ab.20374. 21433031 10.1002/ab.20374

[CR45] Richardson, J. T. (2011). Eta squared and partial eta squared as measures of effect size in educational research. *Educational Research Review*, *6*(2), 135–147. 10.1016/j.edurev.2010.12.001.

[CR47] Rudolph, K. D., Miernicki, M. E., Troop-Gordon, W., Davis, M. M., & Telzer, E. H. (2016). Adding insult to injury: Neural sensitivity to social exclusion is associated with internalizing symptoms in chronically peer-victimized girls. *Social Cognitive and Affective Neuroscience*, *11*(5), 829–842. 10.1093/scan/nsw021. 26892162 10.1093/scan/nsw021 PMC4847705

[CR46] Rudolph, K. D., Davis, M. M., Skymba, H. V., Modi, H. H., & Telzer, E. H. (2021). Social experience calibrates neural sensitivity to social feedback during adolescence: A functional connectivity approach. *Developmental Cognitive Neuroscience*, *47*, 100903. 10.1016/j.dcn.2020.100903. 33370666 10.1016/j.dcn.2020.100903 PMC7773533

[CR48] Ruggieri, S., Bendixen, M., Gabriel, U., & Alsaker, F. (2013). Do victimization experiences accentuate reactions to ostracism? An experiment using cyberball. *International Journal of Developmental Sciences*, *7*(1), 25–32. 10.3233/DEV-1312114.

[CR49] Sambuco, N., Costa, V. D., Lang, P. J., & Bradley, M. M. (2020). Aversive perception in a threat context: Separate and independent neural activation. *Biological Psychology*, *154*(March), 107926. 10.1016/j.biopsycho.2020.107926. 32621851 10.1016/j.biopsycho.2020.107926 PMC7490760

[CR50] Schönberg, T., Daw, N. D., Joel, D., & O’Doherty, J. P. (2007). Reinforcement learning signals in the human striatum distinguish learners from nonlearners during reward-based decision making. *Journal of Neuroscience*, *27*(47), 12860–12867. 10.1523/JNEUROSCI.2496-07.2007. 18032658 10.1523/JNEUROSCI.2496-07.2007 PMC6673291

[CR51] Schreuders, E., Klapwijk, E. T., Will, G. J., Güroğlu, B., & Cognitive (2018). *Affective & Behavioral Neuroscience*, 18, 127–142. 10.3758/s13415-017-0557-1. 10.3758/s13415-017-0557-1 PMC5823968 29318509

[CR52] Schulz, C. C., Von Klitzing, K., Deserno, L., Sheridan, M. A., Crowley, M. J., Schoett, M. J. S., Hoffmann, F., Villringer, A., Vrtička, P., & White, L. O. (2022). Emotional maltreatment and neglect impact neural activation upon exclusion in early and mid-adolescence: An event-related fMRI study. *Development and Psychopathology*, *34*(2), 573–585. 10.1017/S0954579421001681. 35105412 10.1017/S0954579421001681

[CR53] Tops, M., & Boksem, M. A. S. (2011). A potential role of the inferior frontal gyrus and anterior insula in cognitive control, brain rhythms, and event-related potentials. *Frontiers in Psychology*, *2*(330). 10.3389/fpsyg.2011.00330. 10.3389/fpsyg.2011.00330 PMC3212750 22084637

[CR61] Tzourio-Mazoyer, N., Landeau, B., Papathanassiou,D., Crivello, F., Etard, O., Delcroix, N., Mazoyer, B., & Joliot, M. (2002).Automated Anatomical Labeling of Activations in SPM Using a MacroscopicAnatomical Parcellation of the MNI MRI Single-Subject Brain, *NeuroImage*, *15*(1), 273–289. 10.1006/nimg.2001.0978 10.1006/nimg.2001.0978 11771995

[CR54] van der Meulen, M., Steinbeis, N., Achterberg, M., van IJzendoorn, M. H., & Crone, E. A. (2018). Heritability of neural reactions to social exclusion and prosocial compensation in middle childhood. *Developmental Cognitive Neuroscience*, *34*, 42–52. 10.1016/j.dcn.2018.05.010. 29936358 10.1016/j.dcn.2018.05.010 PMC6969304

[CR55] Vijayakumar, N., Cheng, T. W., & Pfeifer, J. H. (2017). Neural correlates of social exclusion across ages: A coordinate-based meta-analysis of functional MRI studies. *Neuroimage*, *153*, 359–368. 10.1016/j.neuroimage.2017.02.050. 28235565 10.1016/j.neuroimage.2017.02.050 PMC5457721

[CR56] Will, G. J., Crone, E. A., & Güroğlu, B. (2015). Acting on social exclusion: Neural correlates of punishment and forgiveness of excluders. *Social Cognitive and Affective Neuroscience*, *10*(2), 209–218. 10.1093/scan/nsu045. 24652858 10.1093/scan/nsu045 PMC4321620

[CR57] Will, G. J., Crone, E. A., Van Lier, P. A. C., & Güroǧlu, B. (2016a). Neural correlates of retaliatory and prosocial reactions to social exclusion: Associations with chronic peer rejection. *Developmental Cognitive Neuroscience*, *19*, 288–297. 10.1016/j.dcn.2016.05.004. 27261927 10.1016/j.dcn.2016.05.004 PMC6988598

[CR58] Will, G. J., van Lier, P. A. C., Crone, E. A., & Güroğlu, B. (2016b). Chronic childhood peer rejection is associated with heightened neural responses to social exclusion during adolescence. *Journal of Abnormal Child Psychology*, *44*(1), 43–55. 10.1007/s10802-015-9983-0. 25758671 10.1007/s10802-015-9983-0 PMC4715124

[CR59] Williams, K. D., Cheung, C. K. T., & Choi, W. (2000). Cyberostracism: Effects of being ignored over the internet. *Journal of Personality and Social Psychology*, *79*(5), 748–762. 10.1037/0022-3514.79.5.748. 11079239 10.1037//0022-3514.79.5.748

