# Supplementary material for: Here Comes Revenge: Peer Victimization Relates to Neural and Behavioral Responses to Social Exclusion
Source: Res Child Adolesc Psychopathol. 2024 Sep 17;52(12):1913–30. doi: 10.1007/s10802-024-01227-4 (PMC11624251; doi:10.1007/s10802-024-01227-4)

**Supplementary materials for manuscript “Here comes revenge:**

**Peer victimization relates to neural and behavioral responses to social exclusion”**

Supplement 1: Utilized questionnaires

Victimization

Provided definition: “Bullying is when some children repeatedly harass another child. Thus, bullying is that you are mean to someone else over and over again. It is difficult for the child who gets bullied to defend itself against this.

Bullying can happen in different ways. For example by …

- Hitting someone, kicking, or pinching
- Steal or damage someone’s belongings
- Make fun of someone, calling names, say mean things
- Gossip about someone
- Exclude someone from games or other activities
- Bullying can also take place via the computer or on the cellphone through text message, WhatsApp, Instagram, Facebook, or snapchat

Bullying is not the same as a fight between two children who are equally strong. Bullying is not teasing as a joke. Bullying is treating someone repeatedly in a mean way.”

1. How often were you bullied in the past months?

I have been bullied:

1. I was called nasty names or laughed in my face or hurt by insults
2. I was left with no attention or outside all things or all company by my classmates
3. I was hit, kicked, pushed, pinched, or spit on
4. Others tried to get others hating me by telling lies about me and gossiping
5. I was cyberbullied: I got nasty or insulting messages, calls, or pictures

Need satisfaction (after each ballgame)

1. I was very confident about myself during the game
2. I felt that I was important

Positive mood (after each ballgame)

1. I felt bad during the game (R)
2. I was happy during the game
3. I was nervous during the game (R)
4. I was sad during the game (R)

Inclusion perception (after MRI scanning, before debriefing)

1. During the first game I was included by the other players
2. During the second game I was included by the other players

Intention to punish

1. I would like to punish [Female excluder] and [Male excluder]
2. I would like to help [Female excluder] and [Male excluder]
3. I would like to hurt [Female excluder] and [Male excluder]
4. I would find it difficult to bring something upon [Female excluder] and [Male excluder]

Supplement 2: Behavioral analyses without adjusted outliers

Regressing (*unadjusted for outliers*) intent to punish on repeated victimization showed that repeated victimization related to intent to punish (*F*(1,80) = 4.06, *p* = .047, *b*(*SD*) = 0.45(0.23), partial η^2^ = .05). The regression of (*unadjusted for outliers*) intent to punish on recent victimization was not significant (*F*(1,82) = 1.99, *p* = .162, partial η^2^ = .02).

**Table S1.** *Statistics of the ROI analyses without adjusted outliers on the relation between neural activity and repeated victimization, neural activity and recent victimization, and neural activity and intention to punish.*

|  | **Repeated victimization** | | | |  | **Recent victimization** | | | |  | **Intention to punish** | | |  |
| --- | --- | --- | --- | --- | --- | --- | --- | --- | --- | --- | --- | --- | --- | --- |
|  | ***F*** | ***df*** | ***p*** | ***η^2^_p_*** |  | ***F*** | ***df*** | ***p*** | ***η^2^_p_*** |  | ***F*** | ***df*** | ***p*** | ***η^2^_p_*** |
| **Exclusion No Ball > Inclusion Ball** | | | | | | | | | | | | | |  |
| Multivariate test | 0.92 | 4,68 | .460 | .05 |  | 1.54 | 4,70 | .200 | .08 |  | 1.36 | 4,70 | .256 | .07 |
| Insula | 3.25 | 1,71 | .076 | .04 |  | 3.93 | 1,73 | .051 | .05 |  | 2.22 | 1,73 | .141 | .03 |
| dACC | 0.11 | 1,71 | .738 | <.01 |  | 0.56 | 1,73 | .458 | .01 |  | 0.05 | 1,73 | .829 | <.01 |
| IFG | 0.59 | 1,71 | .445 | .01 |  | 0.56 | 1,73 | .457 | .01 |  | 0.08 | 1,73 | .779 | <.01 |
| dlPFC | 0.17 | 1,71 | .683 | <.01 |  | 1.00 | 1,73 | .321 | .01 |  | 1.32 | 1,73 | .254 | .02 |
| **Exclusion No Ball > Inclusion No Ball** | | | | | | | | | | | | | |  |
| Multivariate test | 0.97 | 4,68 | .431 | .05 |  | 1.74 | 4,70 | .152 | .09 |  | 2.10 | 4,70 | .090 | .11 |
| Insula | 3.08 | 1,71 | .084 | .04 |  | 4.12 | 1,73 | .046* | .05 |  | 6.25 | 1,73 | .015* | .08 |
| dACC | 0.01 | 1,71 | .942 | <.01 |  | 0.50 | 1,73 | .482 | .01 |  | 0.00 | 1,73 | 1.00 | <.01 |
| IFG | 1.46 | 1,71 | .231 | .02 |  | 1.82 | 1,73 | .182 | .02 |  | 0.12 | 1,73 | .736 | <.01 |
| dlPFC | 0.04 | 1,71 | .835 | <.01 |  | 0.04 | 1,73 | .834 | <.01 |  | 0.63 | 1,73 | .429 | .01 |
| **Inclusion No Ball > Inclusion Ball** | | | | | | | | | | | | | |  |
| Multivariate test | 0.36 | 4,68 | .836 | .02 |  | 0.50 | 4,70 | .737 | .03 |  | 1.45 | 4,70 | .226 | .08 |
| Insula | 0.03 | 1,71 | .866 | <.01 |  | 0.15 | 1,73 | .701 | <.01 |  | 2.48 | 1,73 | .119 | .03 |
| dACC | 0.11 | 1,71 | .738 | <.01 |  | 0.03 | 1,73 | .869 | <.01 |  | 0.08 | 1,73 | .780 | <.01 |
| IFG | 0.40 | 1,71 | .530 | .01 |  | 0.83 | 1,73 | .365 | .01 |  | 0.02 | 1,73 | .902 | <.01 |
| dlPFC | 0.70 | 1,71 | .407 | .01 |  | 1.07 | 1,73 | .305 | .01 |  | 0.18 | 1,73 | .675 | <.01 |

*Note.* **p* < .05, ***p* < .01. Abbreviations: dACC = dorsal anterior cingulate cortex; IFG = inferior frontal gyrus; dlPFC = dorsolateral prefrontal cortex.

**Figure S1.** A) Relation between recent victimization and bilateral insula activity in A) the contrast “Exclusion No Ball > Inclusion Ball” and B) the contrast “Exclusion No Ball > Inclusion No Ball”.


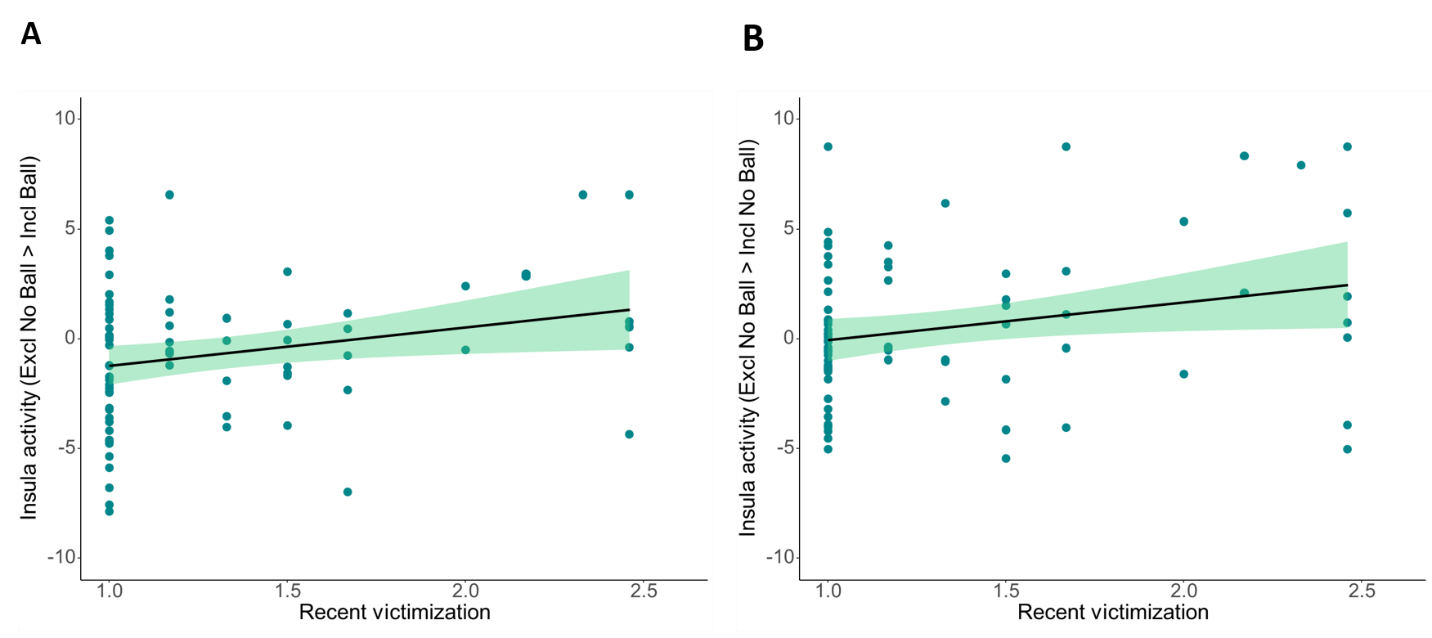


**Figure S2.** Relation between bilateral insula activity in the contrast “Exclusion No Ball > Inclusion No Ball” and intention to punish.


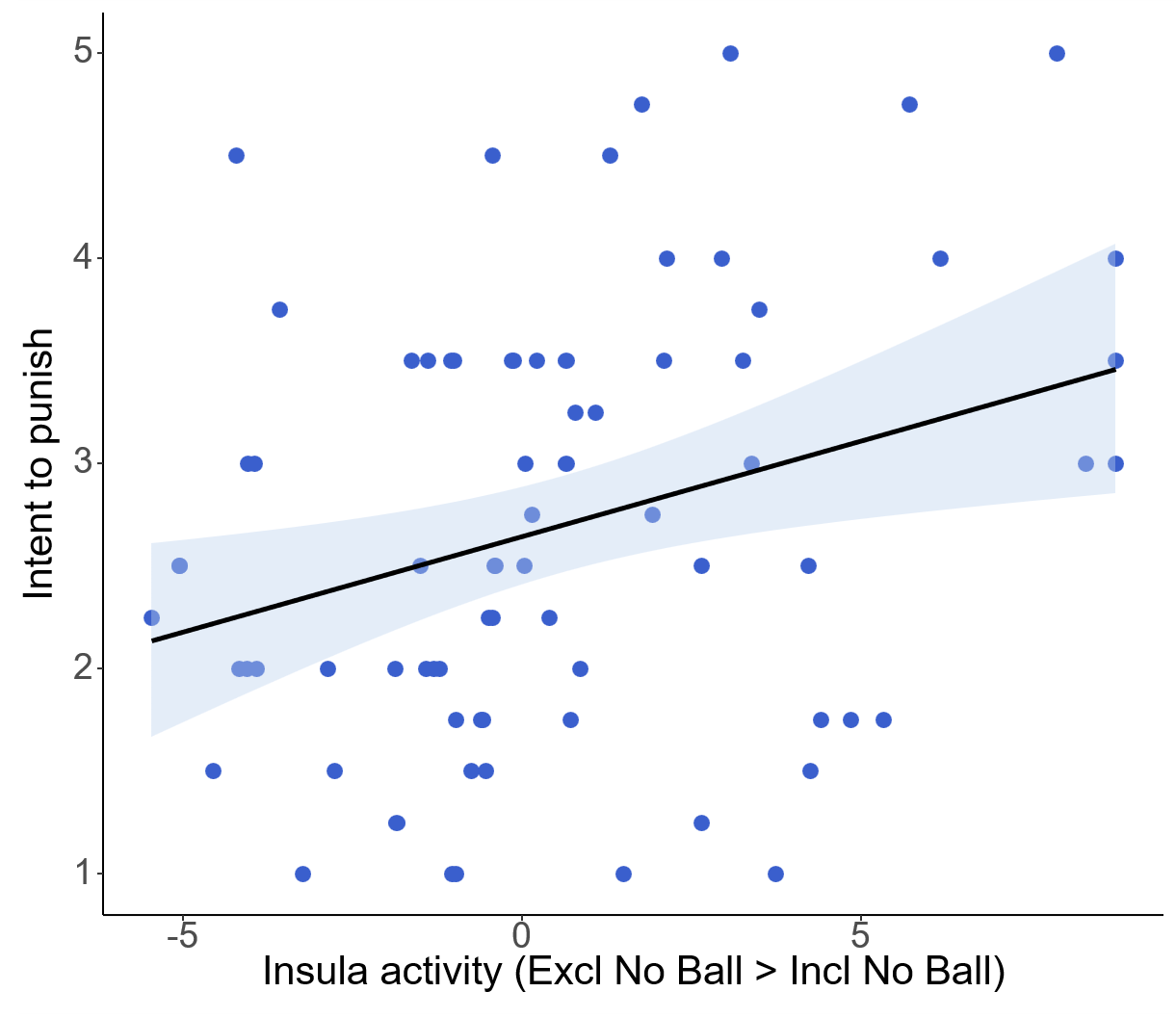

Supplement: Supplementary file 1 — Supplementary Material 1 [file 10802_2024_1227_MOESM1_ESM.docx]
